# Amino Acid-Carbohydrate Intake Combined with Multiple Bouts of Resistance Exercise Increases Resting Energy Expenditure

**DOI:** 10.5402/2013/948695

**Published:** 2013-05-26

**Authors:** Kyle J. Hackney, Andrew R. Kelleher, Lori L. Ploutz-Snyder

**Affiliations:** Department of Exercise Science, Syracuse University, 820 Comstock Avenue, Women's Building 201, Syracuse, NY 13244, USA

## Abstract

Increasing the rate of muscle protein synthesis is an energy consuming process that explains the acute elevations in resting energy expenditure (REE) observed 12 to 72 hours after a resistance exercise session. We hypothesized that multiple sessions of resistance exercise combined with the intake of amino acids would increase REE and alter the nonprotein respiratory exchange ratio (RER). Ten male participants completed two separate seven-day trials where REE and RER were measured on each morning via indirect calorimetry. On four consecutive days within each seven-day trial, acute resistance exercise was performed, and nutritional intake was manipulated by providing (1) amino acids and carbohydrate (AA-RT) or (2) nonnitrogenous, isoenergetic carbohydrate (CHO-RT) before and during each resistance exercise session. Average REE within the training period was 3.61% greater in AA-RT (7897 ± 252 kJ) compared to CHO-RT (7622 ± 289 kJ; *P* = 0.02). RER declined (*P* < 0.0001) from baseline after each resistance exercise was initiated in both AA-RT (0.82 ± 0.01 to 0.77 ± 0.01) and CHO-RT (0.82 ± 0.02 to 0.77 ± 0.02). We conclude the provision of amino acids with multiple bouts of resistance exercise enhances energy expenditure at rest without altering the utilization of lipid.

## 1. Introduction

Total daily energy expenditure is the sum of the thermic effect of feeding, the energy expended during physical activity, and resting energy expenditure (REE). REE represents the largest (i.e., 60–75%) component of total daily energy expenditure [1]. Subsequently, increases in REE can influence daily energy utilization and affect overall energy balance when energy intake is stable [2]. Under normal circumstances there is little day-to-day variability in REE (coefficient of variation (CV) = 1.5–4.0%) [3, 4]. However, an acute bout of heavy resistance exercise has a powerful influence on energy expenditure as it has been shown to elevate REE for 14 to 72 hours after exercise [5, 6]. 

One explanation for the increase in REE in upwards of 24 hours after an acute bout of resistance exercise is increased muscle protein synthesis [7]. The synthesis of muscle proteins is energetically expensive and occurs as a result of the cellular and molecular mechanisms governing mRNA translation. This interaction is one of the most complex activities in the cell as it requires a precise coordination between charged tRNA, amino acids, ribosomes, mRNA, numerous proteins, and energy [8]. Translation elongation appears to require the most energy as four adenosine triphosphate (ATP) equivalent molecules are required for every amino acid added to the peptide chain [9]. Several studies have investigated the acute (<3 hours) changes in energy expenditure during interventions that stimulate muscle protein synthesis. For example, amino acids infused at rates that were known to increase muscle protein synthesis were positively correlated with an increase in REE (*r* = 0.79) [10]. Pre- and/or postresistance exercise intake of amino acids or protein has also been known to increase the fractional rate of muscle protein synthesis [11] and resulted in an elevation of excess postexercise oxygen consumption [12]. Overall, it is estimated that the energy contribution from increased muscle protein synthesis in a highly trained male can be as high as 2030 kJ · day^−1^ [1] and may account for 20% of REE [13]. 

The type of macronutrient metabolized for energy has also been shown to change following an acute bout of resistance exercise. Previous studies have shown that the nonprotein respiratory exchange ratio (RER), an indirect assessment of substrate utilization, declines 10 and 24 hours after an acute resistance exercise bout [14]. Over a longitudinal time frame when muscle protein synthesis is elevated from resistance exercise training and nutritional intake, the increase in lean body mass may be accompanied by a decrease in fat mass [1]. This was demonstrated in a recent study where essential amino acid-carbohydrate supplements were ingested in conjunction with 12 weeks of resistance training in young men [15]. In this study, those who consumed the essential amino acid-carbohydrate after resistance exercise showed greater increases in fat free mass and larger declines in fat mass compared to other subjects that consumed essential amino acids only, carbohydrate only, or a placebo after resistance exercise. These adaptations may be related to the acute energy expenditure and substrate utilization responses in the postexercise period. Understanding these changes could be significant when considering that resistance training on consecutive days reflects the training habits of competitive athletes and recreational exercisers. Therefore, the purpose of this investigation was to examine how multiple bouts of resistance exercise with and without the strategically timed intake of amino acids affect REE and RER. It was hypothesized that intake of amino acids with each resistance exercise session would lead to greater perturbations of REE and RER.

## 2. Materials and Methods

### 2.1. Participants

 Ten male participants (mean ± SD, 23.4 ± 2.5 yr, 175.3 ± 9.7 cm, 77.73 ± 13.95 kg, and 14.4 ± 6.2% body fat) were recruited for the study. All participants were recreationally trained, which was defined as having participated in general resistance exercise for a minimum of 3 days per week for at least 6 months prior to initial testing. Prior to participation, all participants read and signed a written informed consent form that was approved by the Institutional Review Board at Syracuse University. 

### 2.2. Preliminary Testing

Height was measured to the nearest 0.10 cm using a wall mounted stadiometer. Body mass was measured to the nearest 0.01 kg using the electric scale within the BOD POD system (Life Measurement Inc., Concord, CA, USA). Body density was estimated from the measurement of body volume using air displacement plethysmography via the BOD POD [16]. Body fat percentage was calculated accordingly using the Siri equation [17]. One repetition maximum (1RM) strength was determined on three free weight exercises (squats, bench press, and weighted dumbbell lunges) and eight machine exercises (lat pulldown, shoulder press, leg extension, leg curl, biceps curl, triceps extension, seated calf raise, and weighted abdominal twists) in the same order. A research assistant determined the success or failure of each attempt and recorded the final weight that was successfully lifted as the 1RM. Because a 1RM test could not be performed for sit-ups, a sit-up endurance test was performed by counting the maximum number of sit-ups a participant could properly perform in one minute. The results of the 1RM and abdominal endurance tests were used to set exercise prescription during two trials where participants performed four acute bouts of upper and lower body resistance exercise with a seven-day period.

### 2.3. Experimental Design

**Figure 1:**
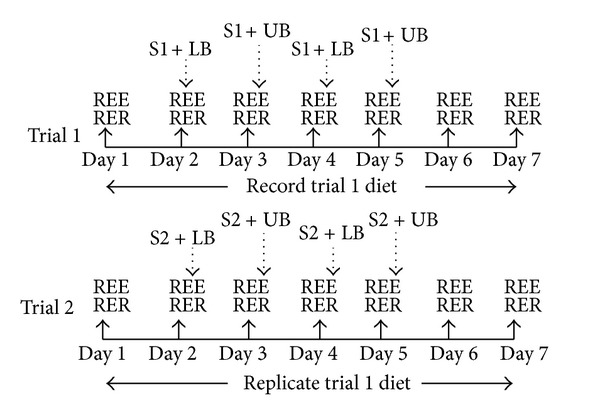
Experimental design. S1: supplement one (randomly assigned and counterbalanced); S2: supplement two (supplement not provided in S1); LB: lower body resistance exercise; UB: upper body resistance exercise.

All participants completed two seven-day trials where REE and RER were obtained each morning, and four resistance exercise sessions were completed (Figure 1). Therefore, participants served as their own control because they participated in both trials. On the first day of each trial no resistance exercise bouts were performed, and only metabolic measures were obtained. Resistance exercise was implemented after metabolic measurements on days 2, 3, 4, and 5. On the second and fourth days, three sets of lower body exercises were performed to volitional fatigue. The exercises were performed in the following order: squats, dumbbell lunge, hamstring curl, leg extension, calf raise, and sit-ups. On the third and fifth days, three sets of upper body exercises were performed to volitional fatigue. The exercise was performed in the following order: bench press, lat pulldown, shoulder press, biceps curls, triceps extension, and weighted abdominal twists. The intensity of each resistance exercise was set at ~75% of their predetermined 1RM. Exercise prescription for sit-ups was determined by calculating a target repetition goal of ~75% of the maximum number of sit-ups performed on the abdominal endurance test. One hundred and twenty seconds of rest was allowed between sets for bench press and squats, while 90 seconds was allowed for all other exercises. On days six and seven only metabolic measures were obtained. 

During each trial, nutritional intake with resistance exercise was manipulated by providing (1) amino acids and carbohydrate (AA-RT) or (2) nonnitrogenous, isoenergetic carbohydrate only (CHO-RT) using a double-blind protocol. Assignment of AA-RT or CHO-RT was randomly assigned and counterbalanced, such that an equal number of participants performed AA-RT in their first trial and an equal number performed CHO-RT in their first trial. The amino acid beverage (Twinlab: Amino Fuel; 22 g protein (6 g essential amino acids: L-phenylalanine: 633 mg; L-valine: 781 mg; L-tryptophan: 133 mg; L-threonine: 679 mg; L-isoleucine: 565 mg; L-methionine: 292 mg, L-histidine: 282 mg; L-leucine: 1350 mg; L-lysine: 1449 mg)) dosage was chosen because 6 g essential amino acids had previously shown to increase muscle protein synthesis in the postresistance exercise period [11]. The amino acids beverage was mixed with a sports recovery drink (36 g carbohydrate, 0 g fat) to increase palatability and minimize any potential gastrointestinal discomfort. CHO-RT was a sports recovery beverage that contained 58.5 g carbohydrate, 0 g fat, and 0 g protein. Both AA-RT and CHO-RT ingested beverages were mixed with 800 mL of cold water by a research assistant that was not involved in supervising the resistance exercise sessions or performing metabolic data collection or analyses. Of this mixture, 400 mL was ingested immediately prior (<5 min) to the resistance training session, while the remaining was consumed during the rest periods between sets during the resistance exercise workout at the leisure of the participant. There was a slight color difference between the nutritional beverages; therefore, aluminum foil was placed over each beverage container, and participants were instructed not to discuss the taste of the beverages with the researcher during exercise sessions.

Nutritional intake outside of resistance exercise sessions during each training period was recorded by having participants document their food and beverage consumption during each trial using a dietary journal. Prior to the first training period, participants were educated on dietary intake recording including the proper documentation of serving sizes of foods and beverages by a member of the research team. Additionally, participants were encouraged to maintain their normal dietary habits over the course of the first training period (except for experimentally manipulated intake). After the first training period, the journals were collected by the research team and then returned to each participant prior to starting the second training period. In the second training period, participants replicated the diet they previously recorded in the first training period. Total energy and macronutrient intake were analyzed using nutritional software (The Food Processor SQL Nutritional Analysis Software from ESHA Research, Salem, OR, USA).

### 2.4. Metabolic Testing

Resting metabolism was measured via indirect calorimetry during each seven-day trial (0630 hr) using recommendations described previously [18]. Briefly, CO_2_ and O_2_ were measured using a ventilated hood method connected to a SensorMedics (Vmax Series 2900) metabolic system. Prior to testing, participants adhered to the following conditions: (1) refrained from alcohol and caffeine for 24 hours and 12 hours, respectfive, and (2) avoided eating or drinking anything but water for 8 hours prior to testing. Immediately before each test, the metabolic system was calibrated in a temperature stable room (20°C–24°C) for gas and flow using known gas concentrations and a 3L syringe. Participants rested supine for 10–15 minutes prior to having the canopy placed over their head. During metabolic testing, participants remained supine and minimized movement for 15–20 minutes. A Polar Heart Rate Monitor was used to monitor heart rate during each minute of the testing period. The first 5 minutes of data was removed via a laboratory standard procedure with metabolic testing. REE was determined by averaging 15 minutes of steady state data [18]. Fat and carbohydrate oxidation were determined indirectly by monitoring the nonprotein RER (VCO_2_ · VO_2_^−1^). Nonprotein RER was determined by averaging data from the same 15-minute period used to determine REE. All metabolic analyses were performed by one investigator that was blinded to the nutritional intervention.

### 2.5. Statistical Analyses

Exercise volume (kg lifted · number of sets · repetitions completed) on each day of exercise was compared between AA-RT and CHO-RT using paired Student's *t*-tests. All metabolic and resting heart rate measures obtained on the first two mornings (before experimental manipulation) were averaged and served as baseline preintervention variables (no significant differences were observed in all variables (*P* > 0.05)). Separate 2 × 6 (condition by time) ANOVAs with repeated measures were performed for each variable (REE, RER, heart rate, nutritional intake). Significance was set at *α* = 0.05. Post hoc time effects were further explored using the Bonferroni correction for multiple comparisons.

## 3. Results

**Figure 2:**
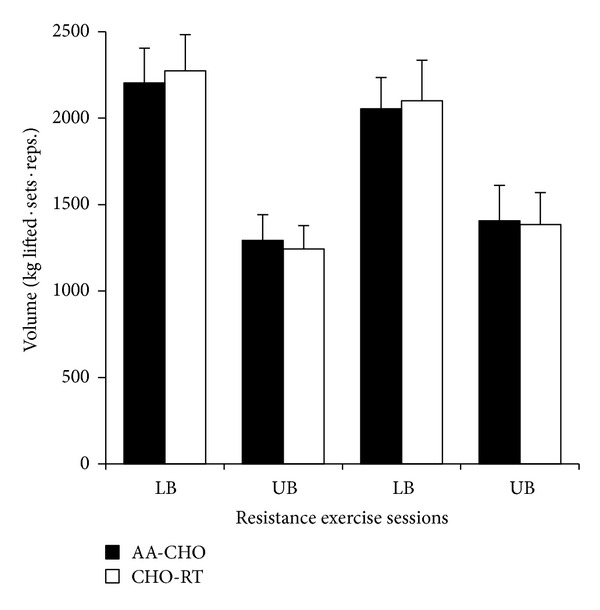
Resistance exercise volume (kg lifted · sets · repetitions) in AA-RT and CHO-RT. LB: lower body resistance exercise; UB: upper body resistance exercise. No differences were detected between AA-RT and CHO-RT for each LB and UB resistance exercise sessions. Data are mean ± SE; *P* > 0.05.

**Figure 3:**
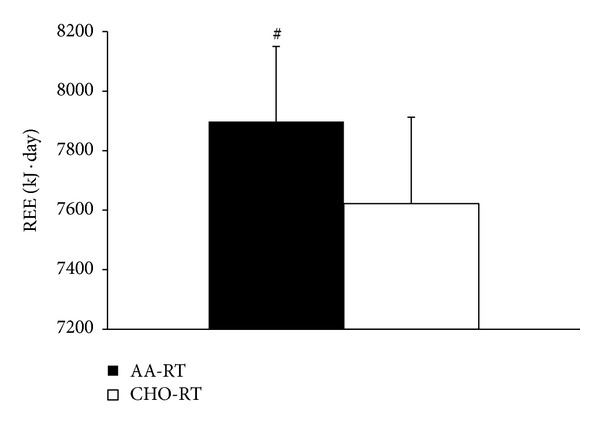
REE condition effect. ^#^Average REE during each 7-day trial was significantly greater in AA-RT versus CHO-RT. Data are mean ± SE; *P* < 0.05.

**Figure 4:**
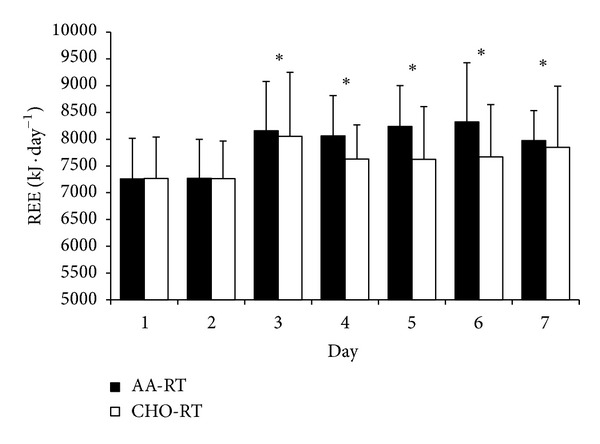
REE time effect. *REEs on days 3, 4, 5, 6, and 7 in both AA-RT and CHO-RT were significantly greater than baseline (average of days 1 and 2). Data are mean ± SE; *P* < 0.05.

**Figure 5:**
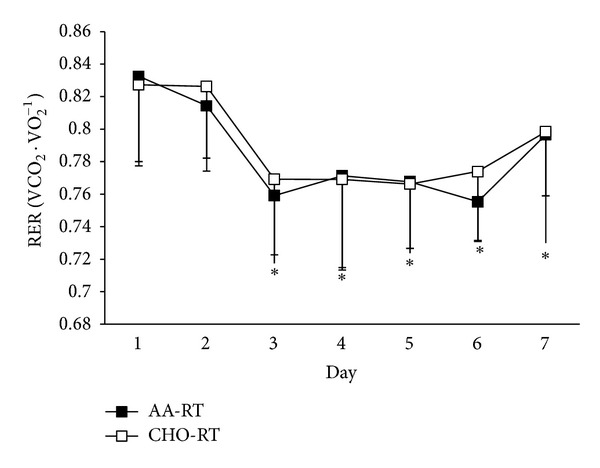
RER time effect. *RERs on days 3, 4, 5, 6, and 7 are significantly less in both AA-RT and CHO-RT than baseline (average of days 1 and 2). Data are mean ± SE; *P* < 0.05.

On average, one month separated each seven-day training period for each participant (30 ± 9 days). There were no differences in resistance exercise volume (indicator of performance) between AA-RT and CHO-RT on any training day (Figure 2, *P* > 0.05). Total energy intake was not different between AA-RT (132.09 ± 15 kJ · kg^−1^ · day^−1^) and CHO-RT (136.26 ± 14 kJ · kg^−1^ · day^−1^) during each training period (*P* = 0.443). However, there were differences in macronutrient composition due to the nutritional manipulation with resistance exercise (Table 1). For REE, there were significant condition (*P* = 0.028) and time (*P* < 0.001) effects. The significant condition effect which determined average REE during the 7-day trial was greater in AA-RT compared to CHO-RT (Figure 3). The significant time effect showed that REE was increased significantly from baseline on each morning after multiple resistance exercise sessions were initiated regardless of condition (Figure 4). A significant time effect was also observed for RER (*P* < 0.001), which was decreased significantly from baseline on each morning after resistance exercise sessions were initiated regardless of condition (Figure 5). There were no differences in resting heart rate between (*P* = 0.411) or within (*P* = 0.08) conditions (Table 2). 

## 4. Discussion

The main finding of this investigation was over the course of a seven-day period REE was 3.61% higher when amino acids were consumed with resistance exercise. REE represents the largest component of total daily energy expenditure (60–85%) and has been implicated as a major contributor to overall body mass management [2]. For example, average REE in the AA-RT training period was equivalent to 7897 kJ per day (1886 kcals · d^−1^) compared to 7622 kJ per day (1820 kcals · d^−1^) in CHO-RT. The net difference represented an additional 275 kJ (66 kcals) of energy expended each day at rest in the AA-RT. If this is extrapolated across the seven-day training period, the AA-RT would have utilized 1934 kJ (462 kcals) more energy than CHO-RT. These data suggest that one benefit of consuming amino acids with resistance exercise, in addition to muscle hypertrophy with consistent training, is the acute elevation in energy expenditure which is likely related to the process of muscle growth. Although the net change in REE on a given day (66 kcals) is small, over time, subtle elevations in REE may help with body composition management.

 Although the current study was not designed to understand the precise mechanisms responsible for the observed changes in REE, we can speculate into how these alterations may occur. Within the first few hours after ingesting amino acids and performing resistance exercise, the fractional rate of skeletal muscle protein synthesis is enhanced by increasing the delivery and uptake of amino acids into skeletal muscle, thereby activating cell-signaling cascades that facilitate increased rates of mRNA translation [11]. The synthesis of muscle proteins is energetically expensive as four ATP molecules are utilized for every amino acid incorporated into a growing peptide chain [9]. However, the anabolic response from timing amino acid intake with resistance exercise is not limited to the first few hours of recovery. Insulin-like growth factor I (IGF-1), a known activator of muscle protein synthesis via the PI3K-Akt-mTOR signaling pathway [19], has a delayed secretion where peak values may not be observed until 16–28 hours after resistance exercise [20]. In a recent study, IGF-1 concentrations were elevated on days 2 and 3 of a three-day paradigm when protein and carbohydrate were ingested before and after each resistance exercise session [21]. Therefore, it is plausible that a basal hormonal environment in favor of muscle protein synthesis may provide a mechanism for how REE may be elevated 24 hours after amino acid intake is coupled with heavy resistance exercise. 

Alternatively, the present study does not indicate that there is any additional benefit from the amino acid intake with resistance exercise on total body fat oxidation. Previous studies have shown that RER (indicator of lipid utilization) is reduced for as long as 24 hours following an acute bout of resistance training [2, 14]. It was hypothesized that AA-RT would provide more of an anabolic response compared to CHO-RT, which would result in a corresponding increase in energy expenditure and lipid utilization (because lipid is the primary fuel source at rest). Our data clearly demonstrated that RER decreased the morning following each resistance exercise session in both conditions. This was evident when both upper and lower body resistance training protocols were utilized. However, we could not detect any differences between AA-RT and CHO-RT conditions. Therefore, it appears that acute resistance exercise performed on multiple days facilitates increased fat utilization, although ingestion of amino acids does not enhance the response. 

## 5. Conclusion

In summary, all participants completed four acute bouts of resistance exercise (alternating lower and upper body) within a seven-day period during two separate trials. During one trial, acute resistance exercise was combined with amino-acid and carbohydrate intake. In the second trial, acute resistance exercise was combined with carbohydrate alone. Indicators of resting metabolism (REE and RER) were measured before the resistance exercise and nutritional interventions began and on each morning after the resistance exercise and nutritional interventions were initiated. We conclude the additional provision of amino acids with multiple bouts of acute resistance exercise enhances energy expenditure at rest without altering the utilization of lipid. 

## Figures and Tables

**Table 1:** Macronutrient intake.

	AA-RT	CHO-RT	*P*-value
Protein (g·kg^−1^·d^−1^)	1.71 ± 0.18^#^	1.49 ± 0.17	0.042
Carbohydrate (g·kg^−1^·d^−1^)	3.43 ± 0.40	3.73 ± 0.41	0.093
Fat (g·kg^−1^·d^−1^)	1.21 ± 0.16	1.23 ± 0.16	0.843

Data are mean ± SD; ^#^significantly greater versus CHO-RT.

**Table 2:** Resting heart rate during metabolic testing.

	AA-RT	CHO-RT
Day 1	58 ± 8	57 ± 6
Day 2	57 ± 7	58 ± 7
Day 3	61 ± 9	60 ± 7
Day 4	60 ± 6	58 ± 6
Day 5	60 ± 7	59 ± 7
Day 6	59 ± 7	58 ± 6
Day 7	58 ± 8	58 ± 7

Data are mean ± SD. All *P* values > 0.05.
